# The relationship between drinking alcohol and esophageal, gastric or colorectal cancer: A nationwide population-based cohort study of South Korea

**DOI:** 10.1371/journal.pone.0185778

**Published:** 2017-10-03

**Authors:** Yoon Jin Choi, Dong Ho Lee, Kyung-Do Han, Hyun Soo Kim, Hyuk Yoon, Cheol Min Shin, Young Soo Park, Nayoung Kim

**Affiliations:** 1 Department of Internal Medicine and Seoul National University Bundang Hospital, Seongnam, Gyeonggi-do, South Korea; 2 Department of Internal Medicine and Liver Research Institute, Seoul National University College of Medicine, Seoul, South Korea; 3 Department of Biostatistics, College of Medicine, The Catholic University of Korea, Seoul, South Korea; University Hospital Llandough, UNITED KINGDOM

## Abstract

**Background:**

Epidemiologic findings of low-volume alcohol consumption in relation to gastrointestinal cancers including gastric cancer are inconsistent.

**Methods:**

The association between alcohol intake and esophageal, gastric and colorectal cancer risk was examined in a population-based prospective cohort of 23,323,730 adults in Korea who had undergone a biennial evaluation provided by the National Health Insurance Corporation between the years 2009 and 2012. After median 5.4 years of follow-up, 9,171 esophageal, 135,382 gastric and 154,970 colorectal cancer cases were identified. Cox regression models were used to estimate hazard ratios (HR) and corresponding 95% confidence intervals (95% CI).

**Results:**

Light drinking as well as moderate to heavy alcohol consumption significantly increased the risks of the three gastrointestinal cancers (HR 1.51; 95% CI, 1.43–1.60; HR 1.08; 95% CI, 1.06–1.09; HR 1.12; 95% CI, 1.11–1.14) compared with non-drinkers after adjusting for age, sex, smoking, exercise, income, body mass index, and diabetes. The synergistically increased cancer risk between excessive amount of alcohol consumption and currently smoking or underweight individuals was observed only in the esophageal cancers.

**Conclusions:**

Light drinking including even one alcoholic drink a day is associated with increased risks of esophageal, gastric and colorectal cancer.

## Introduction

Alcohol is one of the most well-established causes of cancer. A study published in 2011 found that alcohol is responsible for around 4% of cancers in the UK [1]. There is convincing evidence that alcohol consumption increases the risk of cancer in the colorectum, female breast, larynx, liver, esophagus, oral cavity and pharynx [2]. However, much of the existing data are based on Western populations [3,4]. Because the drinking pattern and type of alcoholic beverages consumed are different between Asia and the West, more data from Asia is required. Furthermore, among the Asian populations, the prevalence of the variant allele of aldehyde dehydrogenase-2, which breaks down acetaldehyde to acetate in the metabolism of alcohol, is much higher (28–45%) in comparison with other ethnic groups [5].

Although a large volume of alcohol consumption is generally considered to be linked with various cancer risks, few studies have investigated the association of light and moderate alcohol consumption [6,7], which are the most prevalent levels of alcohol consumption, with total cancer risk. The effect of light or moderate alcohol consumption on carcinogenesis is controversial [8–10]. Finally, the evidence is less clear for a possible association between alcohol consumption and the development of stomach cancer while excessive amount of alcohol consumption has been regarded as a risk factor for esophageal or colorectal cancers.

In Korea, more than 95% of the esophageal cancer types are the squamous cell-type [11]. Stomach cancer is still the most common cancer in Korea except for thyroid cancer [12], and the incidence rate of colorectal cancer in Korea has become the highest in the world exceeding that of the U.S [13].

In this context, we analyzed cohort data from the National Health Insurance Corporation in South Korea in order to determine whether alcohol consumption has an influence on esophageal, gastric and colorectal cancer risks according to prediagnostic alcohol consumption patterns. We also evaluated the effect of alcohol cessation on the risk for the three gastrointestinal cancers.

## Methods

### Data source and study population

The database of the National Health Insurance Corporation (NHIC) was used, which is managed by the Korean government. In this database, approximately 97% of the Korean population is enrolled [14] which receives a semicompulsory biennial medical examination. Any researcher can use the NHIC database if the study protocols are approved by the official review committee, and he or she pays for the data.

**Fig 1:**
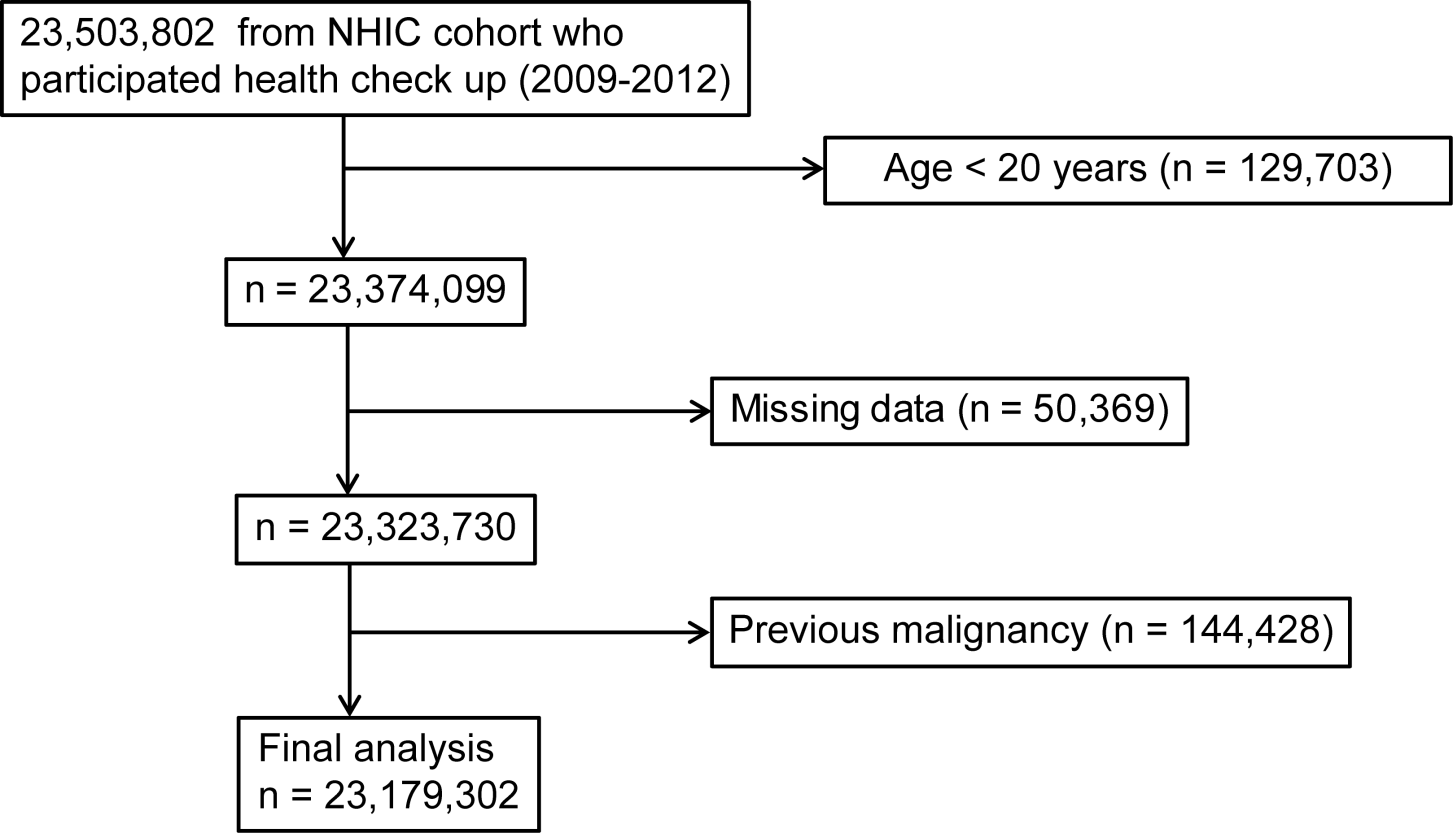
Flowchart showing the enrolment process for the study cohort. NHIC, National Health Insurance Corporation.

Among 23,503,802 individuals who had undergone a biennial or annual evaluation provided by the NHIC between the years 2009 and 2012, data were evaluated from the medical records of 23,323,730 individuals aged 20 years and over. The primary endpoint of this study was newly diagnosed esophageal, gastric, and colorectal cancers, which were defined with the International Classification of Diseases, 10th revision (ICD-10) codes (C150-155, C158, C159, C160 except for C1699 and C180-200) and the National Cancer Registry database. To avoid enrolling patients with pre-existing diseases, individuals diagnosed with esophageal, stomach and colorectal cancer during the preceding year were excluded. Fig 1 shows a summary of the selection of the study population.

### Data collection

Standardized self-reporting questionnaires were used to collect data to establish a baseline for the following variables which are regarded as risk factors for gastric cancer and were included as covariates in the multivariate analyses: age (years), sex, residency (rural and urban), yearly income (lower quintile vs. the remaining quintiles), cigarette smoking (never, former, and current), and physical activity level (low, moderate, or high). BMI and systolic and diastolic blood pressure (mmHg) were also measured. Values for total cholesterol (mg/dL) and liver enzymes including ALT, AST and GGT in serum (IU/L) were determined.

The frequency of alcohol consumption in 1 week and the amount of alcohol consumed on one occasion were evaluated by reviewing the questionnaire; (frequency: 0–7 days/week and amount: drinks per occasion).

Without a standardized definition of alcohol consumption for light and heavy drinking [15], we developed a consumption classification criterion based on the amount of pure alcohol consumed per day in the study population. Alcohol consumption per occasion was surveyed based on 360 ml of soju with an alcohol concentration of 20%. The study participants were divided into three groups: non-drinkers, mild to moderate drinkers, and heavy drinkers. Participants whose alcohol consumption was less than 30 g per day were defined as mild to moderate drinkers while participants whose alcohol consumption was 30 or more grams per day were defined as heavy drinkers in the total cohort population.

The presence of hypertension was defined if when the presence of at least one claim per year for the prescription of antihypertensive agent under ICD-10 codes I10–I15 was confirmed. Dyslipidaemia was defined when at least one claim per year for the prescription of anti-dyslipidemic agent under ICD-10 codes E78 was documented [16].

Because the study involved routinely collected data, informed consent was not specifically obtained for this study. The study was approved by the Institutional Review Board of Seoul National University Bundang Hospital (X-1608/360-904).

### Statistical analyses

Data are presented as the mean ± SD for normally distributed continuous variables and as proportions for categorical variables. The Student’s t-test and ANOVA were used to analyze continuous variables, and the differences between nominal variables were compared with the chi-square test. The incidence rates of cancers were calculated by dividing the number of events by person-time at risk. To determine the independent association of the amount of alcohol consumption and drinking frequency with the risk of cancer incidence, the Cox regression model was used after adjusting for age, sex, smoking status, exercise, monthly income, diabetes, and BMI. Statistical analyses were done with SAS version 9.4 (SAS Institute, Cary, NC, USA) and R version 3.2.3 (The R Foundation for Statistical Computing, Vienna, Austria, http://www.Rproject.Org). The authors conducted Bonferroni correction for multiple comparisons. A two-sided p-value of less than 0.05 was considered statistically significant.

## Results

### Demographic characteristics

Table 1 shows the demographics of the study population according to the alcohol consumption. Among a total of 23,179,312 study subjects, 2.6% (*n* = 53.5%) were classified as non-drinkers, 38.8% (*n* = 8,994,551) as moderate-drinkers, and 7.7% (*n* = 1,788,073) as heavy drinkers (Table 1). The alcohol user groups were generally younger and included more men. The heavy alcohol consumption group included individuals who smoked more frequently, had higher BMI levels, higher blood pressure/fasting glucose levels, and a higher serum total cholesterol than that of non-drinkers.

**Table 1:** Demographics of the alcohol drinkers and nondrinkers in the NHIC cohort (2009–2012).

	Non	Light to moderate	Heavy
Variables	(*N* = 12,396,688)	(*N* = 8,994,551)	*(N* = 1,788,073)
Sex (Male)	3,891,988 (31.4)	6,205,022 (68.99)	1,648,742 (92.21)
Age (y, mean±SD)	51.1 ± 14.6	43.2 ± 13.0	45.2 ± 12.8
Age over 50 y	6,772,881 (54.63)	2,815,518 (31.3)	662,966 (37.08)
Regular exercise	2,057,692 (16.6)	1,676,994 (18.64)	354,799 (19.84)
Lower quintile of yearly income	2,845,624 (22.95)	1,722,882 (19.15)	333,234 (18.64)
BMI >25 kg/m^2^	3,813,677 (30.76)	2882022 (32.04)	735,021 (41.11)
Diabetes	1,266,967 (10.22)	666,589 (7.41)	213,047 (11.91)
Hypertension	3,518,118 (28.38)	2,001,935 (22.26)	574,975 (32.16)
Dyslipidemia	2,738,495 (22.09)	1,395,369 (15.51)	336,342 (18.81)
Metabolic disorder	5,189,000 (41.86)	2,994,337 (33.29)	801,268 (44.81)
Smoking			
Non-smoker	10,011,526 (80.76)	40,043,66 (44.52)	329,589 (18.43)
Ex-smoker	988,177 (7.97)	1,711,799 (19.03)	418,921 (23.43)
Current-smoker	1,396,978 (11.27)	3,278,383 (36.45)	1,039,563 (58.14)
BMI, kg/m2	23.6±3.3	23.7±3.2	24.4±3.2
Sr glucose, mg/dL	97.3±23.4	96.9±22.4	102.5±28
SBP, mmHg	121.6±15.6	122.1±14.6	126.9±14.8
DBP, mmHg	75.2±10	76.5±10	79.7±10.1
Sr cholesterol, mg/dL (mean±SD)	195.8±37.7	193.1±35.8	196.1±37.2
Esophageal cancer	3,232 (0.03)	3,663 (0.04)	2,176 (0.12)
Gastric cancer	73,419 (0.59)	47,515 (0.53)	14,448 (0.81)
Colorectal cancer	89,613 (0.72)	50,974 (0.57)	14,281 (0.80)
F/U duration	5.4±1.1	5.4±1.1	5.4±1.1

*BMI*, body mass index; *SBP*, systolic blood pressure; *DBP*, diastolic blood pressure; *F/U*, follow up; *Sr*, serum

Metabolic disorder: presence of any of the following: hypertension, diabetes or dyslipidemia

After a median follow-up period of 5.4 years, 3,232 (0.03%), 3,663 (0.04%), and 2,176 (0.12%) individuals developed esophageal cancer in each group. Additionally, 73,419 (0.59%), 47,515 (0.53%), and 14,448 (0.81%) individuals and 89,613 (0.72%), 50,974 (0.57%), and 14,281 (0.80%) individuals developed gastric and colorectal cancer in each group, respectively.

#### Risk of gastrointestinal cancer in subjects stratified according to the amount of alcohol consumption

A multivariate analysis was done controlling for age, sex, regular exercise, income, BMI, diabetes mellitus and smoking status (Table 2). After controlling the above mentioned variables, alcohol consumption was clearly associated with an increased risk of esophageal, gastric and colorectal cancer in a roughly dose-dependent manner (esophageal cancer: HR 1.52 and 3.13, stomach cancer: HR 1.05 and 1.24, and colorectal cancer: HR 1.12 and 1.32 in mild to moderate drinkers and heavy drinkers, respectively).

**Table 2:** Risk factors and adjusted hazard ratios of esophageal, stomach and colorectal cancers.

	Hazard ratio (95% CI)		
Covariate	Esophageal cancer	Stomach cancer	Colorectal cancer
Alcohol consumption ^a^			
Non	1(reference)	1(reference)	1(reference)
Mild to moderate	1.52 (1.45–1.60)	1.05 (1.04–1.06)	1.12 (1.10–1.13)
Heavy	3.13 (2.95–3.32)	1.24 (1.21–1.26)	1.32 (1.30–1.35)
Smoking			
Non	1(reference)	1(reference)	1(reference)
Ex	1.29 (1.21–1.37)	1.22 (1.20–1.24)	1.18 (1.16–1.20)
Current	1.87 (1.77–1.98)	1.34 (1.31–1.36)	1.11(1.10–1.13)
Age (per 1y)	1.10 (1.09–1.10)	1.07 (1.07–1.07)	1.06 (1.06–1.06)
Sex (male)	0.15 (0.14–0.16)	0.48 (0.48–0.49)	0.77 (0.76–0.78)
Exercise (no)	1.08 (1.03–1.14)	1.06 (1.05–1.07)	1.06 (1.05–1.07)
Lower quintile of yearly income (no)	1.17 (1.12–1.23)	1.01 (0.99–1.02)	1.01 (1.00–1.02)
BMI (per 1Kg/m^2^)	0.92 (0.92–0.93)	1.00 (1.00–1.00)	1.02 (1.01–1.02)
Diabetes mellitus (no)	1.15 (1.09–1.21)	1.17 (1.15–1.19)	1.23 (1.21–1.24)

*BMI* body mass index *CI* confidential interval *y* year

^a^ Mild to moderate: ≥10g and < 30g alcohol/day; Heavy ≥ 30g alcohol/day

^b^ adjusted for age, sex, exercise, income, BMI, diabetes mellitus, and smoking status

Current and ex-smokers, ageing, male gender and diabetes mellitus were also risk factors for the three gastrointestinal cancers. In particular, the lower quintile of yearly income significantly elevated the risk of esophageal cancer. In regard to the body mass index (BMI), each 1.0 kg/m^2^ increase of BMI was associated with an 8% decreased risk of esophageal cancer, while each 1.0 kg/m^2^ increase of BMI was associated with a 2% increased risk of colorectal cancer. Regular exercise was associated with the elevated risk of the three gastrointestinal cancers.

In order to evaluate if light drinking is associated with the increased risk for the three gastrointestinal cancers, we conducted further analyses with more segmented ranges of the amount of alcohol consumption. The adjusted hazard ratios of all of the three cancers in those who consumed < 10 g per day (light drinking) were an adverse relationship (S1 Table). The risk for gastric and colorectal cancer increased until the daily amount of alcohol consumption reached 20g. However, after 20g per day, a dose dependent increase was not observed while the risk for esophageal cancer continuously increased. Among the three gastrointestinal cancers, esophageal cancer was the most associated with alcohol consumption as well as with the amount of alcohol consumption.

#### The impact of amount of alcohol consumption on the risk for gastrointestinal cancer according to sex, age, BMI and smoking status

The risk factors for each cancer stratified by consumed alcohol amount for sex, age, BMI and smoking status are listed in Table 3. Generally, the risks of the three cancers increased when the amount of alcohol consumption increased except for the risk of colorectal cancer in women. Men who consumed excessive amount of alcohol showed a higher risk for esophageal, gastric and colorectal cancer than women who were heavy drinkers.

**Table 3:** Subgroup analysis of the risk of esophageal, stomach and colorectal cancers according to alcohol consumption.

Subgroups	Alcohol amount^a^	Hazard ratio (95% CI) ^b^		
		Esophageal cancer	Stomach cancer	Colorectal cancer
Male	Non	1 (reference)	1(reference)	1(reference)
	Mild to moderate	1.55 (1.47–1.63)	1.06 (1.05–1.08)	1.18 (1.16–1.20)
	Heavy	3.17 (2.99–3.37)	1.26 (1.24–1.29)	1.40 (1.37–1.43)
Female	Non	1 (reference)	1 (reference)	1 (reference)
	Mild to moderate	1.16 (0.95–1.41)	1.00 (0.97–1.02)	0.98 (0.96–1.01)
	Heavy	2.45 (1.50–4.03)	1.06 (0.95–1.20)	0.95 (0.87–1.04)
BMI <18.5	Non	1.22 (1.06–1.41)	0.90 (0.86–0.93)	0.78 (0.75–0.82)
	Mild to moderate	2.55 (2.21–2.95)	1.11 (1.05–1.18)	1.03 (0.97–1.09)
	Heavy	3.98 (3.28–4.83)	1.47 (1.34–1.61)	1.46 (1.32–1.61)
BMI 18.5–22.9	Non	1 (reference)	1 (reference)	1 (reference)
	Mild to moderate	1.66 (1.54–1.78)	1.08 (1.06–1.11)	1.12 (1.10–1.14)
	Heavy	3.57 (3.28–3.88)	1.33 (1.29–1.37)	1.38 (1.34.1.43)
BMI 23.0–24.9	Non	0.80 (0.74–0.88)	1.03 (1.01–1.05)	1.06 (1.04–1.08)
	Mild to moderate	1.11 (1.02–1.21)	1.06 (1.04–1.09)	1.18 (1.16–1.21)
	Heavy	2.46 (2.22–2.74)	1.25 (1.20–1.29)	1.38 (1.33–1.43)
BMI 25.0–29.9	Non	0.78 (0.72–0.86)	1.04 (1.02–1.06)	1.10 (1.08–1.11)
	Mild to moderate	1.03 (0.94–1.12)	1.07 (1.04–1.09)	1.23 (1.20–1.25)
	Heavy	1.93 (1.73–2.14)	1.20 (1.17–1.24)	1.40 (1.36–1.45)
BMI≥30.0	Non	0.66 (0.51–0.85)	1.07 (1.02–1.11)	1.13 (1.09–1.17)
	Mild to moderate	0.74 (0.55–0.99)	1.03 (0.97–1.09)	1.23 (1.17–1.30)
	Heavy	1.19 (0.81–1.75)	1.11 (1.01–1.22)	1.23 (1.12–1.35)
Non	Non	1 (reference)	1 (reference)	1 (reference)
smoker	Mild to moderate	1.43 (1.31–1.56)	1.07 (1.05–1.09)	1.12 (1.10–1.14)
	Heavy	3.64 (3.25–4.08)	1.35 (1.29–1.40)	1.44 (1.38–1.49)
Ex	Non	1.30 (1.18–1.42)	1.24 (1.22-.1.27)	1.19 (1.17–1.22)
smoker	Mild to moderate	1.95 (1.79–2.12)	1.30(1.27–1.33)	1.34 (1.31–1.36)
	Heavy	4.11 (3.70–4.55)	1.46 (1.41–1.51)	1.56 (1.51–1.61)
Current	Non	1.87 (1.71–2.04)	1.38 (1.35–1.41)	1.14 (1.12–1.17)
smoker	Mild to moderate	2.96 (2.74–3.20)	1.40 (1.37–1.43)	1.25 (1.23–1.28)
	Heavy	5.58 (5.14–6.06))	1.66 (1.62–1.71)	1.44 (1.40–1.48)

^a^Mild to Mild to moderate: ≥10 g and < 30g alcohol/day; Heavy ≥ 30g alcohol/day

^b^ Adjusted for age, sex, regular exercise, yearly income, diabetes mellitus and, smoking status

In terms of the association of BMI and cancer risk, underweight individuals had an increased risk of esophageal cancer in comparison to other ranges of BMI. Among the non-drinkers, while obesity was associated with a reduced risk for esophageal cancer, individuals with BMI larger than 23.0 Kg/m^2^ were more vulnerable to gastric and colorectal cancer. However, there was no dose-dependent relationship between BMI and gastric and colorectal cancer among those who consumed alcohol.

Further analyses were conducted according to the amount of alcohol consumption and BMI. Generally, there was an increased risk of the three gastrointestinal cancers with an increased amount of alcohol consumption regardless of the BMI range (Table 3). This dose-dependent relationship was the most prominent in esophageal cancer and in those with BMI of less than 18.5 Kg/m^2^. Individuals who were underweight and consumed more than 30g of alcohol, showed a 4.0-fold increase in the risk of esophageal cancer in comparison to non-drinkers with a normal range of BMI (Table 3).

**Fig 2:**
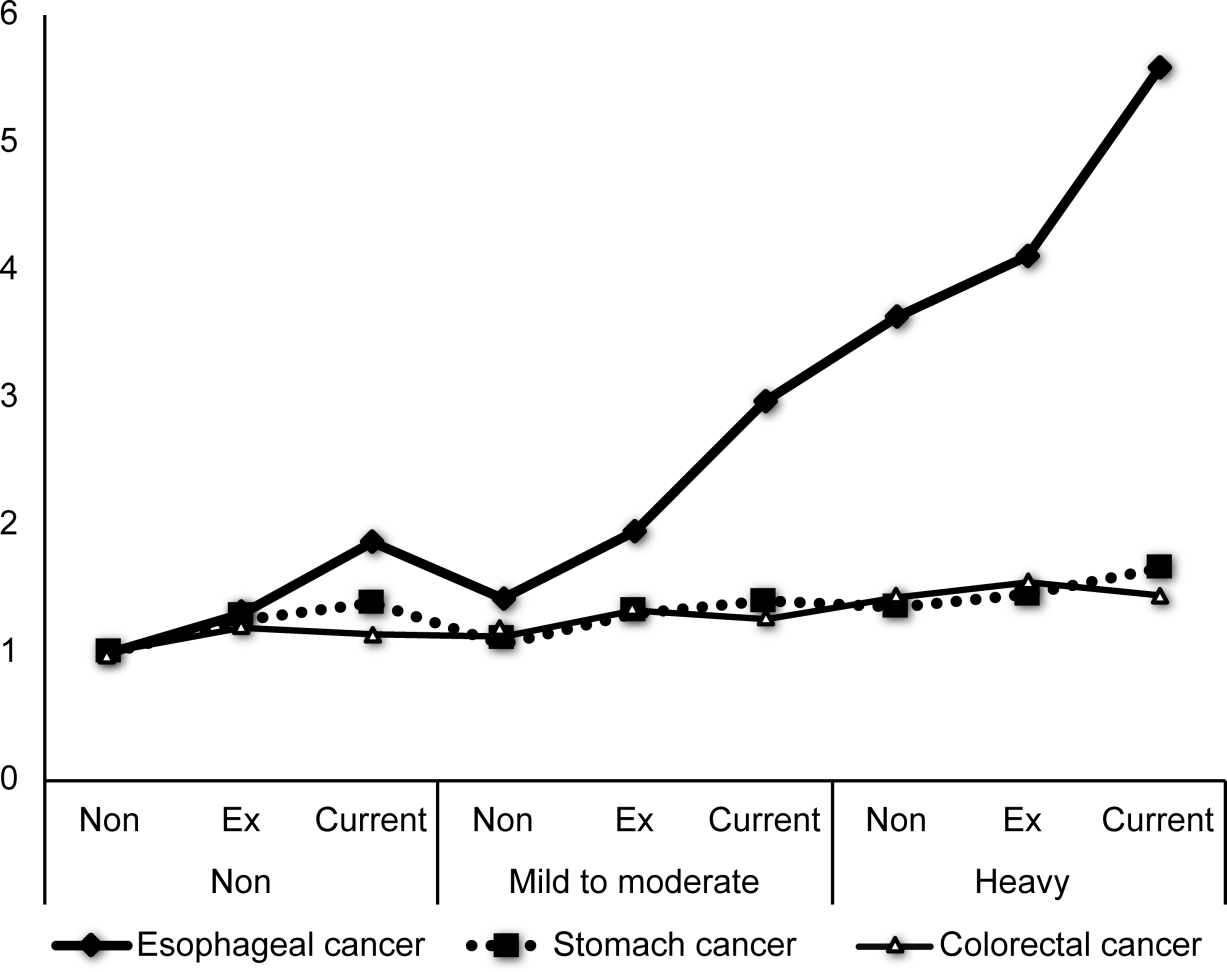
Esophagus, stomach and colorectal cancer risks based on the combined effect of the amount of alcohol consumption and smoking status (A) Esophageal cancer, (B) Gastric cancer, (C) colorectal cancer.

When we evaluated the effect of the amount of alcohol consumption on gastrointestinal cancers according to smoking status, there was a dose-dependent relationship between the amount of alcohol consumption and esophageal, gastric and colorectal cancer regardless of smoking status. However, we observed a significant synergistic effect between excessive alcohol consumption and smoking only in esophageal cancer (HR 5.58, 95% CI 5.14–6.06) (Table 3, Fig 2).

#### Change in the amount of alcohol consumption and developing gastrointestinal cancers

Among the study population, 18,801,552 persons had experienced the follow-up evaluation two years after the baseline. In order to evaluate the effect of change of the amount of alcohol consumption on developing esophageal, gastric and colorectal cancer, we classified participants into nine groups according to the amount of alcohol consumption at the baseline and two-year follow-up (Table 4).

**Table 4:** The effect of change in the amount of alcohol consumption and the risk of developing esophageal, stomach and colorectal cancer.

Alcohol consumption ^a^		No	Event		Duration		IR^b^	HR (95% Cl)
Baseline	2 year F/U							
Esophageal cancer								
1	1	8,900,605	1605	35,488,659			4.52	1 (reference)
	2	1,018,243	258	4,125,435			6.25	1.54 (1.34,1.75)
	3	66,146	79	270,296			29.23	4.09 (3.26,5.14)
2	1	105,1086	333	4,257,820			7.82	1.58 (1.41,1.78)
	2	5,954,491	1716	23,846,387			7.20	1.80 (1.68,1.94)
	3	376,927	242	1,536,391			15.75	3.02 (2.63,3.47)
3	1	74,772	125	304,964			40.99	4.30 (3.58,5.16)
	2	434,004	323	1,766,429			18.29	3.35 (2.97,3.79)
	3	925,278	925	3,690,396			25.07	4.56 (4.18,4.97)
Stomach cancer								
1	1	8,900,605	40216	35,393,686		113.63		1 (reference)
	2	1,018,243	3874	4,117,026		94.10		1.04 (1.00,1.07)
	3	66,146	496	269,282		184.19		1.37 (1.25,1.50)
2	1	1,051,086	4851	4,247,059		114.22		1.10 (1.07,1.13)
	2	5,954,491	23114	23,794,723		97.14		1.11 (1.09,1.13)
	3	376,927	2088	1,532,108		136.28		1.27 (1.21,1.33)
3	1	74,772	685	303,569		225.65		1.36 (1.26,1.46)
	2	434,004	2482	1,761,403		140.91		1.27 (1.22,1.32)
	3	925,278	5736	3,678,240		155.94		1.39 (1.35,1.43)
Colorectal cancer								
1	1	8,900,605	49969	35,380,209		141.23		1 (reference)
	2	1,018,243	4434	4,116,484		107.71		1.09 (1.06,1.13)
	3	66,146	488	269,373		181.16		1.41 (1.29,1.55)
2	1	1,051,086	5528	4,246,334		130.18		1.17 (1.14,1.21)
	2	5,954,491	24851	23,794,897		104.44		1.17 (1.15,1.19)
	3	376,927	2108	1,532,347		137.57		1.34 (1.28,1.40)
3	1	74,772	715	303,636		235.48		1.51 (1.40,1.62)
	2	434,004	2435	1,761,724		138.22		1.30 (1.25,1.36)
	3	925,278	5648	3,679,481		153.50		1.43 (1.30, 1.47)

*CI* confidential interval *F/U* follow up *HR* hazard ratio *IR* incidence rate

a 1 denotes nondrinkers, 2 denotes mild to moderate drinkers and 3 denotes heavy drinkers

^b^ per 100,000

Individuals who had not drunk at the baseline but became current drinkers at the two-year follow-up, showed a higher incidence of esophageal, gastric and colorectal cancer than those who remained a non-drinker in a dose dependent manner. However, the reduction or abstinence of alcohol consumption of persons who had drunk excessively (≥30g/day) did not reduce the risk for the three gastrointestinal cancers. Individuals who consumed less than 30g of alcohol but quit alcohol consumption after the baseline, showed a reduced risk for esophageal cancer. However, individuals who remained current drinkers, still harbored an increased risk in comparison to those who never drank (Table 4).

## Discussion

We showed that prediagnosis alcohol consumption of even less than 10g per day was associated with a significantly higher risk of esophageal (squamous cell-type), stomach, and colorectal cancer in a large cohort of South Korean adults. The risk of esophageal cancer continuously increased in a dose-dependent manner. The risk for gastric and colorectal cancer increased until the daily amount of alcohol consumption reached 20g. However, after 20g per day, a dose dependent increase was not observed. Among the three types of gastrointestinal cancer, esophageal cancer was the most strongly associated with alcohol consumption. The synergistically increased cancer risk between excessive amount of alcohol consumption and currently smoking or underweight individuals was observed only in the esophageal neoplasms.

Alcohol consumption is one of the most important known risk factors for human cancers [17] and simultaneously, one of the most easily avoidable factors. Ethanol from alcoholic beverages is metabolized to acetaldehyde, which was classified as a human carcinogen by the International Agency for Research on Cancer [18]. Acetaldehyde can circulate in the blood after formation in the liver, but can also be formed locally by oral bacteria [19]. Moreover, ethanol itself can cause local irritation of the upper gastrointestinal tract [20] and could also stimulate carcinogenesis by inhibiting DNA methylation.

While higher alcohol consumption was not associated with an increased risk of either esophageal adenocarcinoma or esophagogastric junction adenocarcinoma in a recent meta-analysis [21], a strong relationship with alcohol consumption was observed in esophageal squamous cell cancer and [22–25] in agreement with our result. However, regarding light drinking, there are fewer studies. It has been reported that there was no significant association with esophageal cancer for consumption of less than 170 g/week [10].

It is generally accepted that alcohol consumption could increase risk of developing colorectal cancer [3], but the quantification of the association for light (≤ 1 drink/day) and moderate (2–3 drinks/day) alcohol drinking and the dose–response relationship was not clear. While, there is a report that moderate amount of alcohol consumption compared with little or no alcohol consumption was associated with a reduced risk of colorectal cancer [8], a recent meta-analysis showed no association between light alcohol consumption and colorectal cancer risk [3].

While the association between light or moderate alcohol consumption and esophageal and colorectal cancer is inconclusive, the relation of drinking with stomach cancer has been far less evaluated in comparison with other two cancers. It has been reported that alcohol consumption is not associated either with gastric cardia or non-cardia adenocarcinoma [22] and other two Western cohorts also showed a null effect of alcohol on all types of gastric cancers [24,26]. However, Tramacere et al. [27] reported a positive association only when excessive amount of alcohol (> 50 g/day) was consumed and a higher relative risks for gastric non-cardia cancer than for gastric cardia cancer. Because most of the evidence on the alcohol–cancer link was derived from studies that focused on excessive alcohol consumption, evidence of an association between low levels of alcohol consumption and cancer in the present study is of note. We showed that alcohol consumption less than 10 g per day increased the risk of developing esophageal, stomach and colorectal cancer. This result supports the American Cancer Society Guidelines on Nutrition and Physical Activity for Cancer Prevention which recommends that current drinker restrict the alcohol consumption to 2 drinks per day for men and 1 drink per day for women [28].

The difference in frequency of polymorphism on the aldehyde dehydrogenase-2 is often addressed as a possible mechanism for the conflicting results in the association between alcohol and the three gastrointestinal cancers [4]. The relatively high prevalence of the variant genotype (poor metabolizer) in Asian populations may account for the stronger association between alcohol and gastric and colorectal neoplasia observed in Asian studies [29–31]. Because the pattern of alcohol consumption can change, analyses of alcohol consumption evaluated at one point in time and health outcomes at a later point in time are not accurate. Former drinkers are misclassified commonly as non-drinkers [32]. Former drinkers and life-time abstainers could include less healthy individuals whose poor health is attributable to quitting alcohol [33], which could result in an unfavorable outcome in this group. Moreover, difference in cancer subtypes including histology or location of cancer may be at least partially attributable to the inconsistent data [4,21,34].

Despite the positive association between alcohol consumption and the 3 types of cancers in the current study, it is unclear why the dose-dependent pattern of increased risk for gastric and colorectal cancers was not seen above a prediagnosis alcohol consumption of 20 g per day (S1 Table). This can be explained by the limited number of subjects who were heavy drinkers compared to those who were moderate drinkers [3]. Other complications might hinder heavy drinker at baseline from consuming excessive amount of alcohol, but this is unlikely because the risk remains at least for 2 years after stop a drinking. This study showed an unexpected adverse effect of exercise for prevention of the three cancers and this might come from recall bias or other compounding factors. Comprehensive studies with more exquisite data on the amount and frequency of alcohol consumption, related nutrient status including folate and vitamin B group, and relevant genetic variation may help solve this question [35].

Because alcohol consumption is the modifiable lifestyle factors, the effect of alcohol cessation was investigated. Although there was no significant risk decline regarding esophageal, stomach and colorectal cancer among former heavy drinkers (who quit drinking after baseline), 2 years would be short for evaluating the effect of the drinking cessation. It has been reported that drinkers stay at increased risk for a decade after cessation of alcohol consumption [36]. However, despite the inaccuracy of questionnaire for the assessment of drinking habit, it is notable that a fairly consistent increase in risk for all of 3 cancers was observed in those who became drinkers. Long term follow-up of those who became abstinent from drinking is required.

The major strength of this study is that it is a large, national, population-based study and cohort data that can be followed, not a cross-sectional study. Our study also describes that even light drinking has an adverse effect. Only a small number of studies have reported the effect of light drinking in different smoking strata. Preliminary data in the effect of alcohol consumption change on esophageal, stomach and colorectal cancers was presented. One of the limitations of this study is that the cohort data did not include information on the histological type, TNM stage or location of the cancers. The follow-up period after alcohol cessation may be too short to expect a risk decline effect. We could not evaluate the relevant polymorphisms or conduct an epigenetic study.

In the molecular biological aspect, alcohol consumption can reduce folate levels, inhibit key enzymes in one-carbon metabolism, and hamper the activity and expression of DNA methyltransferases, all of which lead to aberrant patterns of DNA methylation [37]. More recently, a new field of epidemiology, molecular pathological epidemiology (MPE), where genetic and molecular variation is investigated in relation with interactive effects of environmental influences including lifestyle and dietary factor has emerged [38]. MPE can provide a more precise prevention strategy as well as biological evidence for causality by linking putative etiologic factors to specific molecular biomarkers [39]. For instance, Schernhammer et al. [40] reported that the effect of folate intake and alcohol consumption on colon cancer risk could vary according to LINE-1 methylation level. Some studies have evaluated the association between molecularly distinct colorectal cancer subtypes defined by microsatellite instability, CpG island methylator phenotype and/or BRAF mutation status and alcohol consumption [41]. Further study on the effect of alcohol on the development of gastrointesdtianl cancers which is integrated with the influences of folate status and epigenetic varations is warranted.

## Supporting information

S1 Table Number of cancers and adjusted hazard ratios of the three gastrointestinal cancers according to the amount of alcohol consumed daily. (DOCX)
