# Correction: The relationship between drinking alcohol and esophageal, gastric or colorectal cancer: A nationwide population-based cohort study of South Korea

**DOI:** 10.1371/journal.pone.0197765

**Published:** 2018-05-21

**Authors:** Yoon Jin Choi, Dong Ho Lee, Kyung-Do Han, Hyun Soo Kim, Hyuk Yoon, Cheol Min Shin, Young Soo Park, Nayoung Kim

There is an error in Tables 2 and 3. The values “Mild to moderate” in the footnote "a" in the legends of Tables 2 and 3 are defined incorrectly. Please view the corrected tables here.

**Table 2:** Risk factors and adjusted hazard ratios of esophageal, stomach and colorectal cancers.

	Hazard ratio (95% CI)^b^		
Covariate	Esophageal cancer	Stomach cancer	Colorectal cancer
Alcohol consumption ^a^			
Non	1(reference)	1(reference)	1(reference)
Mild to moderate	1.52 (1.45–1.60)	1.05 (1.04–1.06)	1.12 (1.10–1.13)
Heavy	3.13 (2.95–3.32)	1.24 (1.21–1.26)	1.32 (1.30–1.35)
Smoking			
Non	1(reference)	1(reference)	1(reference)
Ex	1.29 (1.21–1.37)	1.22 (1.20–1.24)	1.18 (1.16–1.20)
Current	1.87 (1.77–1.98)	1.34 (1.31–1.36)	1.11(1.10–1.13)
Age (per 1y)	1.10 (1.09–1.10)	1.07 (1.07–1.07)	1.06 (1.06–1.06)
Sex (male)	0.15 (0.14–0.16)	0.48 (0.48–0.49)	0.77 (0.76–0.78)
Exercise (no)	1.08 (1.03–1.14)	1.06 (1.05–1.07)	1.06 (1.05–1.07)
Lower quintile of yearly income (no)	1.17 (1.12–1.23)	1.01 (0.99–1.02)	1.01 (1.00–1.02)
BMI (per 1Kg/m^2^)	0.92 (0.92–0.93)	1.00 (1.00–1.00)	1.02 (1.01–1.02)
Diabetes mellitus (no)	1.15 (1.09–1.21)	1.17 (1.15–1.19)	1.23 (1.21–1.24)

*BMI* body mass index *CI* confidential interval *y* year

^a^ Mild to moderate: < 30g alcohol/day; Heavy ≥30g alcohol/day

^b^ adjusted for age, sex, exercise, income, BMI, diabetes mellitus, and smoking status.

**Table 3:** Subgroup analysis of the risk of esophageal, stomach and colorectal cancers according to alcohol consumption.

Subgroups	Alcohol amount^a^	Hazard ratio (95% CI) ^b^		
		Esophageal cancer	Stomach cancer	Colorectal cancer
Male	Non	1 (reference)	1(reference)	1(reference)
	Mild to moderate	1.55 (1.47–1.63)	1.06 (1.05–1.08)	1.18 (1.16–1.20)
	Heavy	3.17 (2.99–3.37)	1.26 (1.24–1.29)	1.40 (1.37–1.43)
Female	Non	1 (reference)	1 (reference)	1 (reference)
	Mild to moderate	1.16 (0.95–1.41)	1.00 (0.97–1.02)	0.98 (0.96–1.01)
	Heavy	2.45 (1.50–4.03)	1.06 (0.95–1.20)	0.95 (0.87–1.04)
BMI <18.5	Non	1.22 (1.06–1.41)	0.90 (0.86–0.93)	0.78 (0.75–0.82)
	Mild to moderate	2.55 (2.21–2.95)	1.11 (1.05–1.18)	1.03 (0.97–1.09)
	Heavy	3.98 (3.28–4.83)	1.47 (1.34–1.61)	1.46 (1.32–1.61)
BMI 18.5–22.9	Non	1 (reference)	1 (reference)	1 (reference)
	Mild to moderate	1.66 (1.54–1.78)	1.08 (1.06–1.11)	1.12 (1.10–1.14)
	Heavy	3.57 (3.28–3.88)	1.33 (1.29–1.37)	1.38 (1.34.1.43)
BMI 23.0–24.9	Non	0.80 (0.74–0.88)	1.03 (1.01–1.05)	1.06 (1.04–1.08)
	Mild to moderate	1.11 (1.02–1.21)	1.06 (1.04–1.09)	1.18 (1.16–1.21)
	Heavy	2.46 (2.22–2.74)	1.25 (1.20–1.29)	1.38 (1.33–1.43)
BMI 25.0–29.9	Non	0.78 (0.72–0.86)	1.04 (1.02–1.06)	1.10 (1.08–1.11)
	Mild to moderate	1.03 (0.94–1.12)	1.07 (1.04–1.09)	1.23 (1.20–1.25)
	Heavy	1.93 (1.73–2.14)	1.20 (1.17–1.24)	1.40 (1.36–1.45)
BMI≥30.0	Non	0.66 (0.51–0.85)	1.07 (1.02–1.11)	1.13 (1.09–1.17)
	Mild to moderate	0.74 (0.55–0.99)	1.03 (0.97–1.09)	1.23 (1.17–1.30)
	Heavy	1.19 (0.81–1.75)	1.11 (1.01–1.22)	1.23 (1.12–1.35)
Non	Non	1 (reference)	1 (reference)	1 (reference)
smoker	Mild to moderate	1.43 (1.31–1.56)	1.07 (1.05–1.09)	1.12 (1.10–1.14)
	Heavy	3.64 (3.25–4.08)	1.35 (1.29–1.40)	1.44 (1.38–1.49)
Ex	Non	1.30 (1.18–1.42)	1.24 (1.22-.1.27)	1.19 (1.17–1.22)
smoker	Mild to moderate	1.95 (1.79–2.12)	1.30(1.27–1.33)	1.34 (1.31–1.36)
	Heavy	4.11 (3.70–4.55)	1.46 (1.41–1.51)	1.56 (1.51–1.61)
Current	Non	1.87 (1.71–2.04)	1.38 (1.35–1.41)	1.14 (1.12–1.17)
smoker	Mild to moderate	2.96 (2.74–3.20)	1.40 (1.37–1.43)	1.25 (1.23–1.28)
	Heavy	5.58 (5.14–6.06))	1.66 (1.62–1.71)	1.44 (1.40–1.48)

^a^ Mild to moderate: < 30g alcohol/day; Heavy ≥30g alcohol/day

^b^ Adjusted for age, sex, regular exercise, yearly income, diabetes mellitus and, smoking status.
