# Supplementary material for: The relationship between drinking alcohol and esophageal, gastric or colorectal cancer: A nationwide population-based cohort study of South Korea
Source: PLoS One. 2017 Oct 3;12(10):e0185778. doi: 10.1371/journal.pone.0185778 (PMC5626486; doi:10.1371/journal.pone.0185778)
Supplement: S1 Table — (DOCX) [file pone.0185778.s001.docx]

**S1Table** Number of cancers and adjusted hazard ratios of the three gastrointestinal cancers according to the amount of alcohol consumed daily

| Cancer | Amount  of alcohol (g/day) | Number | Duration  (person-year) | Incidence rate^a^ | HR^b^ (95% CI) |
| --- | --- | --- | --- | --- | --- |
| Esophageal cancer | Non | 3,232 | 67282225 | 4.79 | 1 (reference) |
|  | < 10 | 1,406 | 28272815 | 4.97 | 1.20 (1.12-1.28) |
|  | < 20 | 1,267 | 13419698 | 9.44 | 1.93 (1.81-2.07) |
|  | < 30 | 990 | 7152891 | 13.84 | 2.69 (2.50-2.90) |
|  | ≥ 30 | 2,176 | 9706730 | 22.41 | 3.64 (3.44-3.86) |
| Gastric cancer | Non | 73,419 | 67252462 | 109.17 | 1 (reference) |
|  | < 10 | 24,417 | 28199600 | 86.59 | 1.08 (1.06-1.09) |
|  | < 20 | 14,594 | 13377725 | 109.09 | 1.19 (1.17-1.21) |
|  | < 30 | 8,504 | 7128741 | 119.29 | 1.12 (1.09-1.15) |
|  | ≥ 30 | 14,448 | 9666533 | 149.46 | 1.08 (1.04-1.13) |
| Colorectal cancer | Non | 89,613 | 67225416 | 133.30 | 1 (reference) |
|  | < 10 | 27,052 | 281989656 | 95.93 | 1.08 (1.11-1.14) |
|  | < 20 | 15,125 | 13379532 | 113.05 | 1.25 (1.23-1.27) |
|  | < 30 | 8,797 | 7129830 | 123.38 | 1.10 (1.07-1.13) |
|  | ≥ 30 | 14,281 | 9670518 | 147.68 | 1.04 (1.01-1.08) |

CI- confidential interval

^a^Per 1000 person-year

^b^ Multivariate model adjusted for adjusted for age, sex, smoking, exercise, income, BMI, and diabetes
